# The Knowledge, Attitudes, and Experiences of Traditional Korean Medicine Doctors in the Spontaneous Reporting of Adverse Drug Events: A Cross-Sectional Survey

**DOI:** 10.3390/healthcare13131620

**Published:** 2025-07-07

**Authors:** Mikyung Kim, Hyunkyung Sung, Jiyun Jung, Dongjun Choi

**Affiliations:** 1 Department of Internal Medicine, Dongguk University Ilsan Oriental Hospital, Goyang-si 10326, Republic of Korea; 01mkkim@gmail.com; 2 Department of Education, College of Korean Medicine, Dongguk University WISE Campus, Gyeongju 38066, Republic of Korea; mintypink@naver.com; 3 Department of Biostatistics, College of Medicine, Dongguk University, Goyangsi 10326, Republic of Korea; bestjudy21@gmail.com

**Keywords:** herbal medicine, traditional Korean medicine, traditional Korean medicine doctors, pharmacovigilance, spontaneous reporting, adverse events, survey, questionnaire

## Abstract

**Background and Objectives:** The spontaneous reporting (SR) of adverse drug events (ADEs) is a cornerstone of pharmacovigilance and a critical mechanism for safeguarding patient safety. However, underreporting remains a persistent global challenge. In Korea, despite the widespread use of herbal medicines (HMs), adverse event reports from traditional Korean medicine doctors (KMDs) are remarkably scarce. This study aimed to assess the knowledge, attitudes, and experiences of KMDs regarding SR, identify key barriers, and suggest strategies to strengthen the safety culture within traditional Korean medicine. **Materials and Methods:** A cross-sectional, anonymous online survey was distributed to licensed KMDs registered with the Association of Korean Medicine. The questionnaire collected information on respondents’ sociodemographic characteristics and assessed their knowledge, attitudes, and experiences related to ADE reporting. Descriptive statistics and multivariate logistic regression were used to analyze the associations between the variables. **Results:** Of the 1021 KMDs who completed the survey, the vast majority acknowledged the importance of SR and recognized their role in pharmacovigilance. Nevertheless, only 5% had ever submitted an ADE report. A widespread lack of awareness about the national spontaneous reporting system (SRS), particularly its inclusion of licensed HMs, was evident. Although many respondents expressed support for expanding the scope of SR to cover all HMs, significant gaps in pharmacovigilance knowledge and limited access to relevant training were major barriers. KMDs affiliated with academic institutions or specialist groups showed higher levels of awareness, education, and reporting behavior. **Conclusions:** While KMDs exhibit positive attitudes toward patient safety and understand the importance of SR, their participation in it remains low due to knowledge deficits and insufficient training. Addressing these gaps through targeted education and expanding the national SRS to comprehensively include herbal medicines are essential steps toward enhancing pharmacovigilance and cultivating a proactive safety culture in Korean medicine.

## 1. Introduction

Adverse drug events (ADEs) are a significant cause of prolonged hospitalization, morbidity, and mortality, posing a direct threat to patient safety [1]. Although pre-market clinical trials rigorously assess the safety of new drugs, rare and serious ADEs often only emerge in real-world clinical settings [2]. As such, robust pharmacovigilance systems are essential for the ongoing monitoring of drug safety post-approval [1].

To coordinate global pharmacovigilance efforts, the World Health Organization (WHO) launched the Programme for International Drug Monitoring (PIDM) in 1968 [3]. As of today, over 180 countries participate in this program and have contributed more than 40 million individual case safety reports to VigiBase, the world’s largest pharmacovigilance database [3]. This collaborative data sharing enables timely signal detection, facilitating early regulatory action to mitigate harm and enhance patient protection [1,2].

A cornerstone of pharmacovigilance is spontaneous reporting systems (SRSs), through which healthcare professionals and patients voluntarily report suspected ADEs [1]. Despite their critical role, the effectiveness of SRSs is undermined by widespread underreporting, largely due to a lack of awareness, training, and institutional support [4,5,6]. Globally, it is estimated that only around 6% of actual ADEs are reported [6], delaying risk identification and compromising patient safety [7]. Addressing the barriers to reporting is thus imperative for cultivating a proactive safety culture in healthcare [8].

Recognizing the increasing use of herbal medicines (HMs) worldwide, the WHO has emphasized the urgent need to integrate HMs into national pharmacovigilance frameworks [9]. Although HMs have a long tradition of use, many lack rigorous post-marketing safety evaluations [10]. Consequently, post-market surveillance is essential for ensuring the safe and responsible use of HMs [11]. However, many countries either exclude HMs from their SRSs entirely or include only a limited subset of them [12]. Furthermore, the underreporting of HM-related ADEs is particularly prevalent due to a low perceived risk, a lack of standardization, and patients’ reluctance to disclose HM use to healthcare providers [10,11,12,13].

In Korea, traditional East Asian medicine is widely practiced, and herbal medicines are central to Korean medicine. These are dispensed in a variety of forms, ranging from licensed herbal medicinal products (HMPs) manufactured by pharmaceutical companies to self-prepared herbal medicines (SPHMs), such as decoctions and powders, produced by traditional Korean medicine institutions (KMIs). Despite their prevalence, only a limited number of these—mainly licensed HMPs and select herbal raw materials (HRMs)—are included in the national SRS.

The Korean SRS was established in 1988. ADEs related to the use of high-risk drugs such as anticancer agents, antipyretic analgesics, and contrast media are among the most frequently reported [14]. In Korea, the SRS is operated via an online platform managed by the Korean Institute of Drug Safety & Risk Management (KIDS), accessible to both healthcare professionals and consumers. Despite its accessibility, the reporting interface for healthcare professionals follows international standards and may be less intuitive for use in routine clinical settings.

To encourage spontaneous reporting (SR), the Korean government has been setting up regional drug safety centers (RDSCs) since 2006, with the KIDS overseeing their operations [15]. Although most RDSCs are housed within Western medical institutions or are part of the Korean Pharmaceutical Association, a Korean medicine institution was designated as an RDSC specializing in herbal medicines for the first time in 2020 [15]. However, the current SRS continues to exclude many commonly used SPHMs, thereby limiting comprehensive safety surveillance in Korean medicine [12]. As a result, the ADE reporting by traditional Korean medicine doctors (KMDs) remains very low, and HM-related safety data are scarce [16].

Healthcare professionals’ engagement in pharmacovigilance is closely linked to their knowledge, attitudes, and exposure to training [7,8]. Although a 2007 study explored general stakeholders’ views on HMs and ADEs [17], no research to date has specifically examined the perspectives and practices of KMDs. Given their central role in prescribing HMs, understanding the barriers to SR they face is essential to strengthening patient safety within Korean medicine.

This study aimed to assess the knowledge, attitudes, and experiences of KMDs regarding spontaneous ADE reporting. The findings are expected to inform the creation of targeted strategies for enhancing KMDs’ participation in pharmacovigilance and support the development of a more inclusive and effective safety monitoring system, ultimately contributing to a stronger culture of patient safety in contemporary integrative healthcare.

## 2. Materials and Methods

### 2.1. Study Design and Population

This was a cross-sectional study employing an anonymous online survey. The study population comprised KMD members of the Association of Korean Medicine (AKM) who agreed to participate in and completed this survey and were currently engaged in clinical practice or medical education/research (academia). A KMD was defined as a person who had completed a six-year course at a college of traditional Korean medicine (KM) or a four-year course at a graduate school of KM approved by the government of the Republic of Korea, passed a national examination for KMDs, and obtained a license to practice KM from the Ministry of Health and Welfare.

### 2.2. Questionnaire Development

The questionnaire was developed by the authors with reference to conceptual definitions and survey items used in prior related studies [7,8,17]. It consisted of four sections collecting information on the respondents’ sociodemographic characteristics and knowledge, attitudes, and experience regarding the SR of ADEs. Sociodemographic data were collected using six questions on the respondents’ sex, age group (in their 20s, 30s, 40s, 50s, or over 60 years old), years of experience as a KMD (<5, 5–9, 10–19, or ≥20 years), current employment type (self-employed, employed, or in academia), current workplace (a local clinic, other medical institution, or university/research institution), and experience of completing a training course at a KM teaching hospital (none, an internship, or residency training). There were also four questions on respondents’ knowledge level (K1–4), six on their attitudes (A1–6), and four on their experience with SR and related education (E1–4).

Internal consistency and content validity testing were not performed, as the questionnaire was designed to minimize conceptual overlaps and was based on established frameworks and items from previous studies [7,8,17].

### 2.3. Data Collection

The self-developed questionnaire was uploaded to the online platform provided by the Korean Social Science Data Center (KSDC), and a link to it was sent to 27,407 email accounts registered as KMD members of the AKM as of 21 October 2024. An outline of the survey, along with its purpose and planned use, was provided via email, and data were only collected from members who voluntarily agreed to participate. The survey period lasted for 12 days, from 21 October to 1 November 2024. After its completion, the raw data were downloaded from the KSDC platform in a Microsoft Excel file format.

### 2.4. Statistical Analysis

The statistical analyses were limited to respondents who met the inclusion criteria, excluding those not currently involved in clinical practice or medical education/research (academia). Descriptive statistics of the responses to each item are presented as frequencies and percentages. For questions permitting multiple responses, the frequency and percentage were calculated based on the number of participants who responded. For the questions related to knowledge, attitudes, and experience, odds ratios (OR) and 95% confidence intervals (CIs) were calculated using logistic regression analysis to evaluate the influence of respondents’ sociodemographic characteristics on each response. The results of the univariate and multivariate analyses adjusted for respondents’ sex, age group, clinical experience, employment type, workplace, and training experience are presented. In cases where the independent variables were categorical (age group, clinical experience, and training experience), a trend analysis was performed to determine the trend of the dependent variable with changes to higher categories. For two identical questions with multiple answers, the consistency of the response rate for each choice was estimated using the chi-square test. All the results were analyzed using R statistical software (version 4.1.1; R Core Team, 2021), with statistical significance at *p*-values of <0.05.

### 2.5. Ethical Considerations

Before conducting the survey, the research procedure and planned use of the results were submitted to the Institutional Review Board, and an exemption from review was granted (DUIOH IRB 2024-07-001-001). Written informed consent was not obtained to ensure the anonymity of the respondents.

## 3. Results

### 3.1. Sociodemographic Characteristics of the Respondents

A total of 1040 individuals completed the survey, yielding a response rate of 3.8%. Data from 1021 respondents who met the inclusion criteria were included in the analysis. The participants’ sociodemographic characteristics are presented in Table 1. They were male in 65.4% of cases and most commonly in their 30s (33.0%), followed by their 40s (31.0%). Clinical experience of 10–19 years was the most frequently reported (33.6%). The predominant employment type was self-employment (45.4%), followed by salaried employment (42.9%). The most common workplace was a local KM clinic (62.9%), followed by another medical institution (35.3%). More than half of the respondents (60.5%) were general practitioners with no experience of training at KM teaching hospitals. A total of 12.3% of the respondents had completed a one-year internship, and 27.1% were specialists who had completed a one-year internship and three-year residency.

### 3.2. Knowledge Characteristics

**Figure 1:**
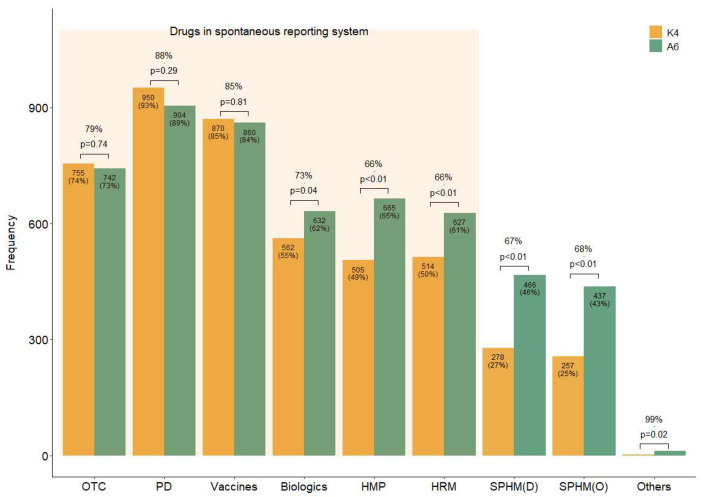
Concordance rates of the responses regarding the drug types thought to be included in the current spontaneous reporting system and those that should be. Note: Answer to questions K4 and A6 in the survey. K4: Which of the following do you believe are currently included in the official ADE targets under the domestic SRS? (Allowing for multiple selections). A6: Which of the following do you believe should be mandatorily included as ADE-reporting targets in domestic SRS? (Allowing for multiple selections). Abbreviations: D, decoction; HMP, licensed herbal medicinal products manufactured by pharmaceutical companies; HRM, medicinal herbs as raw materials; O, other dosage forms than decoction, such as pills or powder, etc.; OTC, over-the-counter drugs; PD, prescription drugs; SPHM, self-prepared herbal medicines by Korean medical institutions.

Four questions (K1–4) were designed to assess the respondents’ knowledge level (Table 2). Overall, 55% of the respondents were aware of the domestic SRS (K1), while 46% were aware that it also included HMs (K2). Additionally, 59% of the respondents answered that they should report ADEs to a regulatory agency even if their causal relationship with a drug is unclear (K3), suggesting that they were aware of the purpose of SR. As illustrated by the K4 bar plot in Figure 1, more than half of the respondents were aware of the drugs included in the current SRS (over-the-counter drugs, prescription drugs, vaccines, biologics, licensed HMPs, and HRMs) (Figure 1 (K4)). However, fewer respondents knew that some HMs (licensed HMPs, 49%; HRMs, 50%) are included in the SRS than those aware of Western medicines’ inclusion (55% versus 93%) (Figure 1 (K4)).

### 3.3. Attitude Characteristics

Six questions (A1–6) were designed to assess the respondents’ attitudes toward SRS (Table 3). The majority of the respondents agreed that KMDs need to actively report ADEs (A1, 88%) and that their role in SRS is important (A2, 89%), showing a positive attitude toward KMDs participating in SR (Table 3).

As shown in Table 4, the most agreed-upon potential outcome of SR (A3) was the accumulation of knowledge on the safe use of drugs (78%), and the three most frequently reported outcomes were all positive. Legal disputes (29%) and heightened social tensions (28%) were the most frequently expected negative outcomes.

The most common reasons for non-participation in SR (A4) were a lack of awareness of the SRS (55%) and reporting procedures (51%), followed by the belief that cases with low causality (46%) and mild AEs (44%) do not need to be reported (Table 5).

The most common measure that would encourage the respondents to engage in SR (A5) was the simplification of the reporting process (63%), followed by strengthening undergraduate education on related tasks (62%) and establishing institutional protocols to resolve legal disputes (56%) (Table 6).

In addition to the drugs already included in the current domestic SRS, respondents believed that it should include all types of HMs, including SPHMs produced by KMIs rather than pharmaceutical companies (A6) (Figure 1). For questions with the same response choices (K4 and A6), the agreement between the response rates for each option was generally high; however, the number of suggestions to include HMs and biologics in the SRS (A6) was significantly higher than the awareness of their current inclusion status (K4) (Figure 1).

### 3.4. Experience Characteristics

Four questions (E1–4) were designed to investigate the respondents’ experiences of SR and related education (Table 7). Half of the respondents (51%) had experienced or witnessed ADEs, but only 5% had reported these to a regulatory agency. Most respondents (91%) had not received any training in pharmacovigilance, including SR procedures or causality assessments. Those with educational experience indicated that they had obtained this through undergraduate study, training courses at teaching hospitals, or continuous education.

### 3.5. Sociodemographic Factors Affecting Respondents’ Knowledge, Attitudes, and Experiences Regarding SR

**Figure 2:**
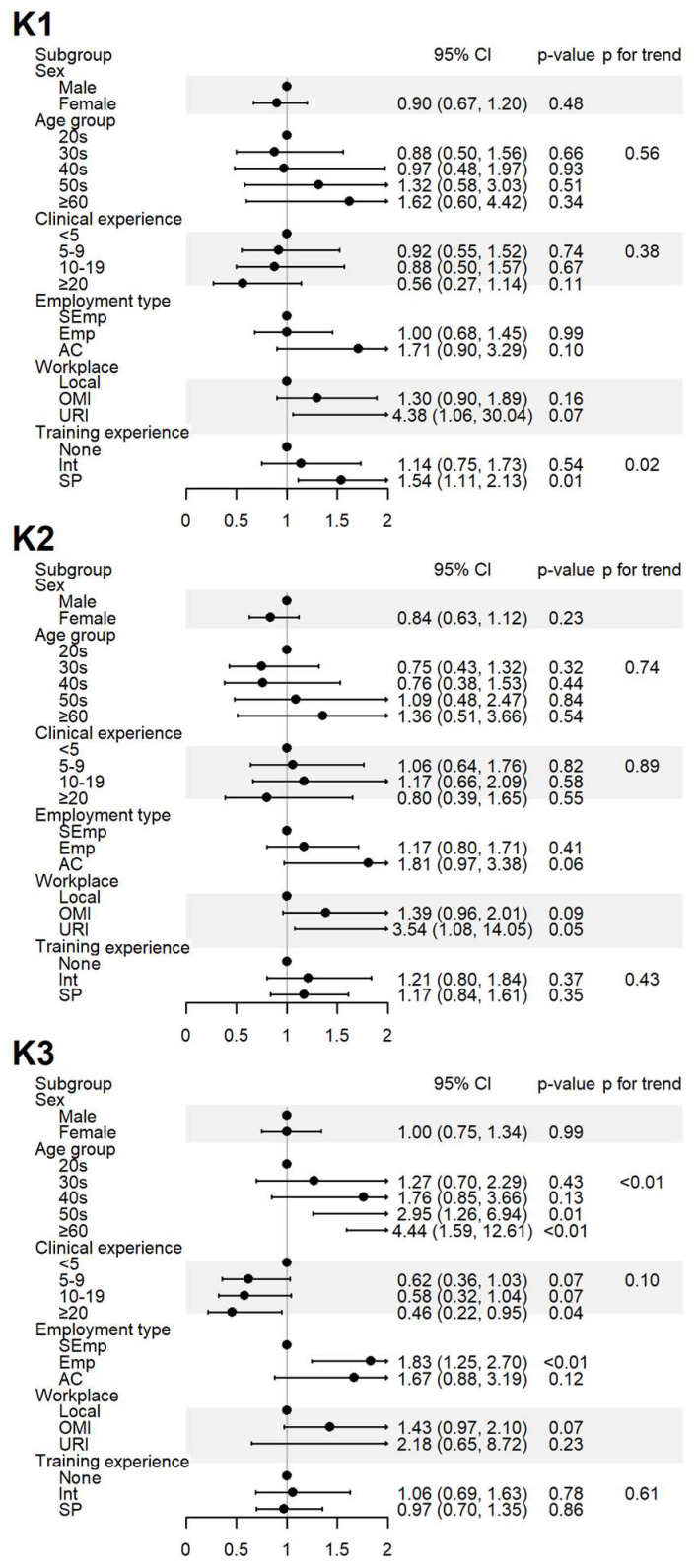
Sociodemographic factors affecting the respondents’ knowledge of spontaneous reporting. K1: I know that ADEs can be reported to the KIDS or RDSCs; K2: I know that ADEs related to HMs can also be reported to the KIDS or RDSCs; K3: I think that ADEs should be reported to the KIDS or RDSCs even if the causal relationship with the drug is uncertain. Abbreviations: AC, academia group engaged in medical education and/or research; CI, 95% confidence interval; Emp, employed KMD; Int, internship; KMD, traditional Korean medicine doctor; Local, local traditional Korean medicine clinic; OMI, other medical institution; OR, odds ratio; *p*, *p*-value; PFT, *p* for trend; SEmp, self-employed KMD; SP, specialist who had completed an internship and residency; URI, university or research institute.

**Figure 3:**
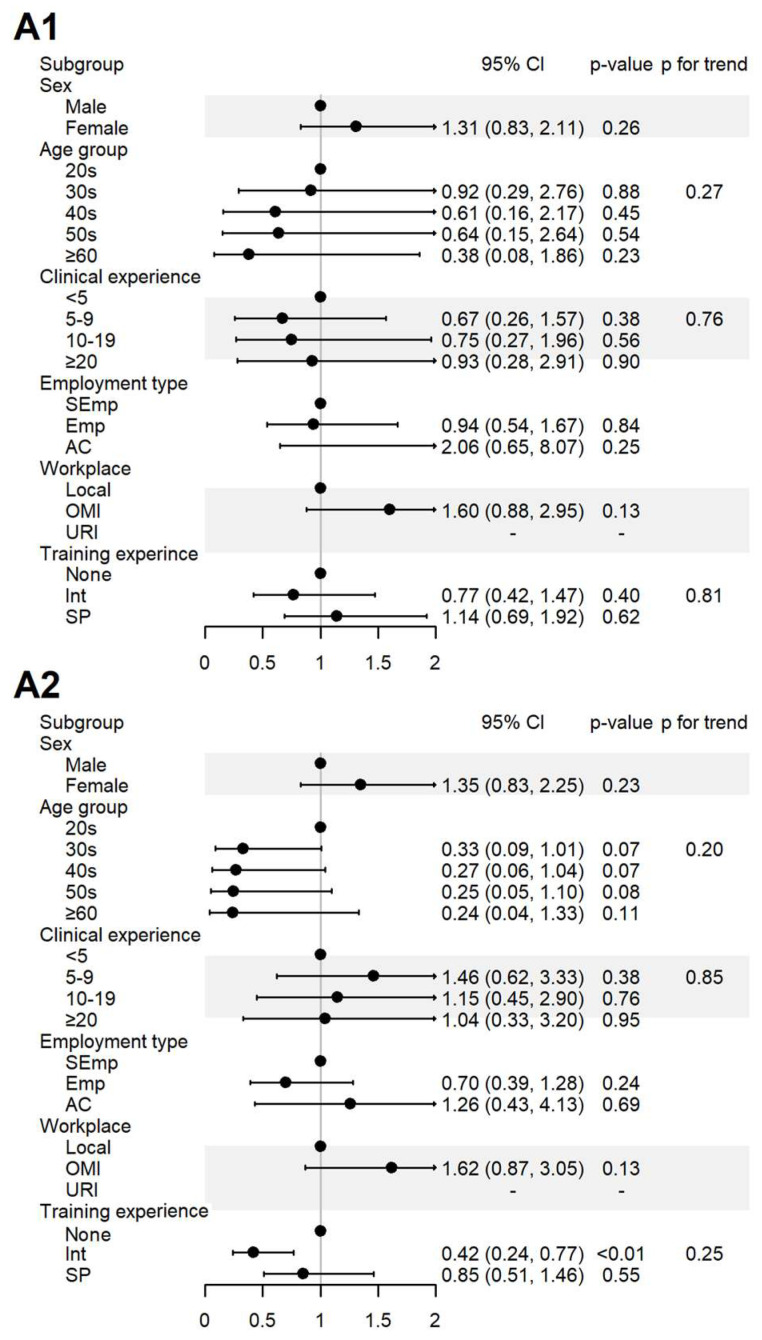
Sociodemographic factors affecting respondents’ attitudes to spontaneous reporting. A1: I agree that KMDs should actively participate in SR; A2: I believe that the role of KMDs is important in SRS. AC, academia group engaged in medical education and/or research; CI, 95% confidence interval; Emp, employed KMD; Int, internship; KMD, traditional Korean medicine doctor; Local, local traditional Korean medicine clinic; OMI, other medical institution; OR, odds ratio; *p*, *p*-value; PFT, *p* for trend; SEmp, self-employed KMD; SP, specialist who had completed an internship and residency; URI, university or research institute.

**Figure 4:**
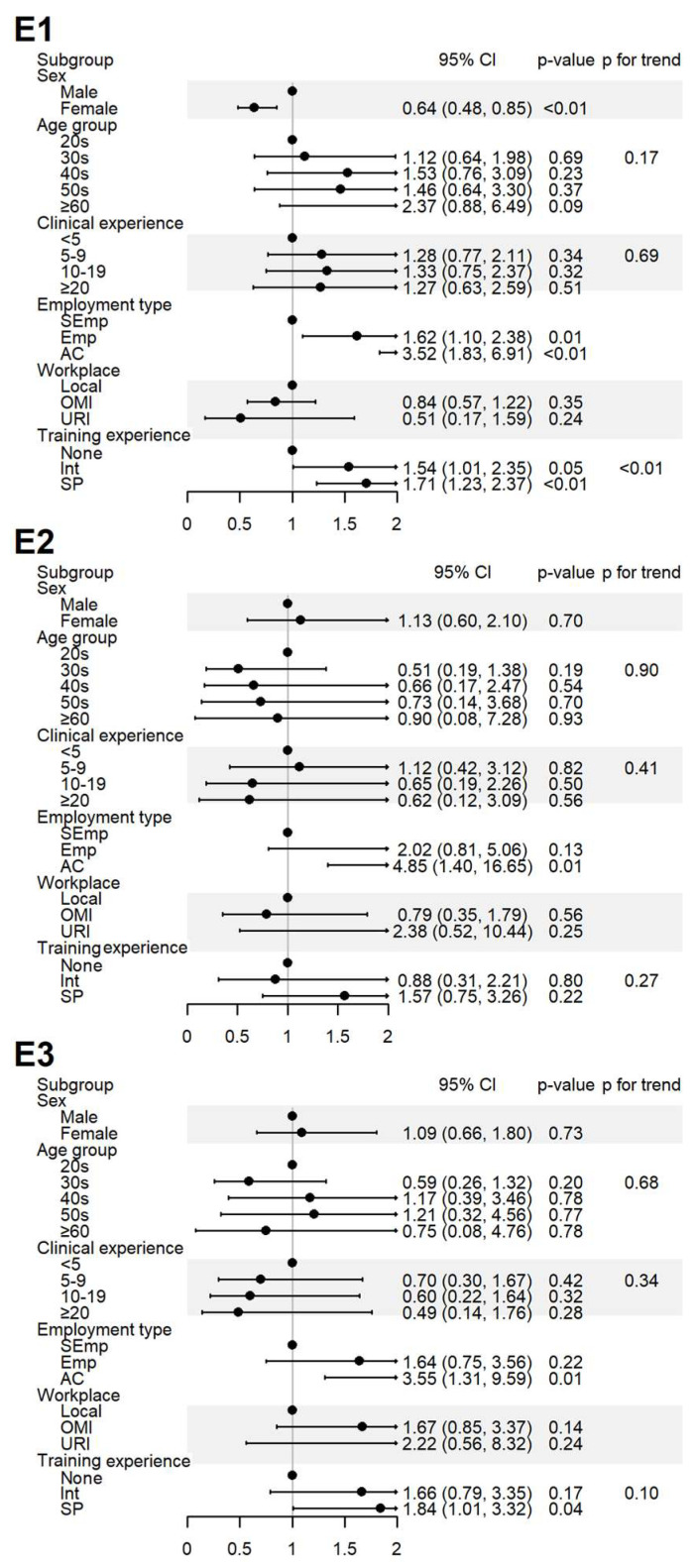
Sociodemographic factors affecting respondents’ experiences of spontaneous reporting. E1: I have never experienced or witnessed an ADE following drug administration; E2: I have never reported an ADE to the relevant authority; E3: I have received training on PV, including SRS procedures and causality assessment. AC, academia group engaged in medical education and/or research; CI, 95% confidence interval; Emp, employed KMD; Int, internship; KMD, traditional Korean medicine doctor; Local, local traditional Korean medicine clinic; OMI, other medical institution; OR, odds ratio; *p*, *p*-value; PFT, *p* for trend; SEmp, self-employed KMD; SP, specialist who had completed an internship and residency; URI, university or research institute.

The impact of the respondents’ sociodemographic characteristics on their responses to yes-or-no questions was assessed. The results are presented in Figure 2, Figure 3 and Figure 4 and Supplementary Table S1.

The level of awareness of the domestic SRS (K1) was significantly higher among specialists, and a positive association was observed with increased training experience. The level of awareness regarding HMs are inclusion in the domestic SRS (K2) showed no significant differences after adjustment. Agreement that ADEs need to be reported to regulatory agencies despite uncertain causality (K3) increased with age and among employed respondents but decreased with greater clinical experience (Figure 2).

For the items assessing the perceived role of KMDs in SR (A1) and its importance (A2), 100% agreement was observed among the respondents affiliated with universities or research institutes. In contrast, the respondents who had only completed internship-level training agreed less with item A2 (Figure 3).

Experience with ADEs (E1) was more frequently reported by male respondents, those employed at institutions, and those with greater training experience. The experience with SR (E2) was significantly higher among respondents in academia. Education in pharmacovigilance (E3) was more common among respondents in academia and specialists (Figure 4).

## 4. Discussion

This study investigated the knowledge, attitudes, and experiences of KMDs regarding the SR of ADEs using an anonymous online survey. Although most KMDs expressed positive attitudes toward SR, the actual reporting rate was very low (5%). The most frequently cited barrier was insufficient knowledge, particularly concerning the national SRS and the existing inclusion of licensed HMPs.

The participants’ responses to the questions on which drug types are currently included (K4) and what should be included (A6) revealed a significant knowledge gap. While KMDs strongly supported the expansion of the SRS to include all HMs, including both licensed HMPs and SPHMs, they were often unaware of the current scope of the system. This discrepancy underscores the urgent need for targeted education and improved communication about the national SRS, as well as its strategic promotion.

In Korea, the Ministry of Food and Drug Safety (MFDS) only includes licensed HMPs and HRMs in its SRS, excluding SPHMs despite their common use by KMDs. Expanding the scope of the SRS to cover all HMs is critical for building a comprehensive pharmacovigilance system. Our findings suggest that KMDs are supportive of this change.

The findings of this study are consistent with the international literature identifying a lack of knowledge (“ignorance”), uncertainty about performing causality assessment (“diffidence”), and the complexity of the reporting process (“lethargy”) as key barriers to reporting [8]. In our study, these same barriers were the most frequently cited.

Notably, a distinctive barrier identified in this context was “fear”—the concern that reporting ADEs could fuel the public criticism or legal scrutiny of HMs. This fear, rooted in interprofessional tension between KM and Western medicine (WM) communities [18], is less commonly present in other healthcare settings. For example, unlike Japan, where traditional medicine has largely been integrated into conventional medicine, or China, where it receives strong governmental support and promotion, Korea maintains a dual medical system in which traditional and conventional medicine operate independently and often compete for patients. This competitive structure, coupled with the overlapping scopes of these practices, has led to more pronounced tensions between the two professions [18].

Against this backdrop, even a single reported ADE following HM use can trigger disproportionate media attention and public criticism, often lacking consideration of the clinical context or a rigorous causality assessment. Such incidents have contributed to persistent skepticism toward HM and the heightened scrutiny of KMDs, reinforcing fears that SR may undermine the perceived safety and legitimacy of both HMs and KMDs. This reluctance persists despite most HMs—whether licensed HMPs or SPHMs—being subject to strict quality control standards and regulatory oversight in Korea. Under such a system, the reported ADEs are likely to be rare and idiosyncratic rather than caused by quality defects such as contamination, misuse, or misidentification [10,11]. Nevertheless, without adequate contextualization, reports are often interpreted as indictments of the entire practice. Therefore, encouraging SR among KMDs requires more than individual-level interventions; broader structural efforts, including government-led initiatives to mitigate interprofessional conflict and improve public trust, are essential for fostering a sustainable safety reporting culture.

Although nearly 90% of the KMDs agreed that their participation in SR is important, only a minority had received formal training, mostly from academic institutions and specialty societies. These groups demonstrated greater awareness, higher reporting rates, and a stronger sense of responsibility, indicating their potential to lead the promotion of SR education and provide mentorship within the KM community.

Educational interventions should go beyond one-time lectures. Research indicates that hands-on, practical training has a greater and more sustained impact on reporting behavior [19,20,21], though these effects may diminish over time without reinforcement [19,22]. Therefore, incorporating structured and recurrent training—starting during undergraduate education and continuing through professional development—is vital to overcoming entrenched barriers.

This requires curriculum reforms at the university level and continuous education efforts led by academic societies and professional associations. Training programs should provide education on practical topics including ADE detection, causality assessment, and step-by-step reporting procedures, as well as the broader societal and clinical value of pharmacovigilance [19,22]. Such efforts may help to address the ignorance, diffidence, and lethargy identified in this study and encourage the greater participation of KMDs in SR.

If SR by KMDs is actively promoted in parallel with improving the herbal pharmacovigilance system, the accumulation of real-world safety data could enable large-scale pharmacoepidemiological research, similar to the analyses conducted using national and international databases [23,24,25].

Finally, several limitations should be acknowledged. This study’s voluntary and self-reported design may have introduced selection and recall biases. Respondents may have had a preexisting interest in pharmacovigilance, and the results may not fully represent all KMDs. Nevertheless, the findings offer valuable insights into the current state of SR within KM and point to actionable paths forward.

To assess the potential selection bias, we compared the demographic and occupational characteristics of our respondents with national data on KMDs. The gender distribution of the respondents (65.4% male, 34.6% female) was broadly consistent with that of all licensed KMDs (approximately 76% male and 24% female) [26]. Participants in their 30s and 40s were the most represented in our sample, which also reflects the dominant age range among practicing KMDs according to recent national statistics [27]. In terms of respondents’ employment type and workplace, 45.4% were self-employed and 62.9% worked in clinics—slightly lower percentages than the national proportions (71% [27] and 78.9%, respectively [28]), but still reasonably representative. However, our sample included a higher proportion of board-certified KM specialists (27.1%) compared with the national data (13% [28]), suggesting that these specialists’ views may have been more prominently reflected. While the sample appeared to be generally representative, the slight overrepresentation of certain subgroups should be considered when interpreting the findings.

## 5. Conclusions

This cross-sectional survey highlights that, despite having generally positive attitudes toward the spontaneous reporting of ADEs, KMDs show low actual participation rates, primarily due to limited knowledge, uncertainty, and the procedural burden. These barriers are similar to those observed internationally but uniquely compounded in Korea by the social and professional tension between KM and WM communities, leading to a fear of criticism and reputational harm.

To increase the SR rates among KMDs, efforts must extend beyond basic awareness campaigns. A multifaceted approach is required—one that improves knowledge, clarifies the scope of the SRS, and addresses interprofessional conflicts and societal distrust toward HMs. Including all HMs in the national SRS and ensuring transparency and fairness in ADE assessment will be essential steps.

Academia and specialist KMDs, while representing a small proportion of the profession, are well-positioned to lead educational initiatives and foster a culture of patient safety. Ultimately, the greater engagement of KMDs in pharmacovigilance will contribute to providing reliable safety data for HMs, thereby supporting their responsible use, improving patient outcomes, and advancing public health in an integrated healthcare system.

## Figures and Tables

**Table 1:** Sociodemographic characteristics of respondents (*n* = 1021).

Characteristics	Categories	No (%)
Sex	Male	668 (65.4%)
	Female	353 (34.6%)
Age group (years)	20–29	103 (10.1%)
	30–39	337 (33.0%)
	40–49	317 (31.0%)
	50–59	215 (21.1%)
	≥60	49 (4.8%)
Clinical experience ^1^ (years)	<5	199 (19.5%)
	5–9	197 (19.3%)
	10–19	343 (33.6%)
	≥20	282 (27.6%)
Employment type	Self-employed KMD	464 (45.4%)
	Employed KMD	438 (42.9%)
	In academia ^3^	119 (11.7%)
Workplace	Local KM clinic	642 (62.9%)
	Another medical institution	360 (35.3%)
	University/research institution	19 (1.9%)
Training experience ^2^	None	618 (60.5%)
	Internship Internship + residency	126 (12.3%)
		277 (27.1%)

Abbreviations: KM, traditional Korean medicine; KMD, traditional Korean medicine doctor. ^1^ Years of experience practicing as a KMD. ^2^ Experience of completing a doctor-in-training course at a KM teaching hospital. ^3^ Engaged in medical education and/or research.

**Table 2:** Respondents’ level of knowledge regarding spontaneous reporting.

Question No.	Questions	Choices	No (%)	*p*-Value
K1	Are you aware that ADEs can be reported to the KIDS or RDSCs?	No Yes	463 (45%) 558 (55%)	<0.01
K2	Are you aware that ADEs related to HMs can also be reported to the KIDS or RDSCs?	No Yes	553 (54%) 468 (46%)	0.01
K3	Do you think that ADEs should be reported to the KIDS or RDSCs even if the causal relationship with the drug is uncertain?	No Yes	421 (41%) 600 (59%)	<0.01
K4	Which of the following do you believe are currently included in the official ADE targets under the domestic SRS? (AMS)	The results are shown in Figure 1.		

Abbreviations: ADE, adverse drug event; AMS, allowing for multiple selections; HM, herbal medicine; KIDS, Korean Institute of Drug Safety; RDSC, regional drug safety center; SRS, spontaneous reporting system.

**Table 3:** Respondents’ attitudes regarding spontaneous reporting.

Question No.	Questions	Choices	No (%)	*p*-Value
A1	Do you agree that KMDs should actively participate in SR?	No Yes	121 (12%) 900 (88%)	<0.01
A2	Do you believe that the role of KMDs is important in SRS?	No Yes	108 (11%) 913 (89%)	<0.01
A3	Which of the following do you agree with are the potential outcomes of SR? (AMS)	The results are shown in Table 3.		
A4	What are the reasons for not reporting ADEs in the past? (AMS)	The results are shown in Table 4.		
A5	Which of the following measures do you think are appropriate for promoting SR by KMDs? (AMS)	The results are shown in Table 5.		
A6	Which of the following do you believe should be mandatorily included as ADE reporting targets in the domestic SRS? (AMS)	The results are shown in Figure 1.		

Abbreviations: ADE, adverse drug event; AMS, allowing for multiple selections; KMD, Korean medicine doctors; SR, spontaneous reporting; SRS, spontaneous reporting system.

**Table 4:** Respondents’ predictions regarding potential outcomes of spontaneous reporting (multiple selections allowed) (*n* = 1021) *.

Choices	No (%)
Knowledge for safe use of drugs is accumulated.	798 (78%)
Improves patient safety.	734 (72%)
Increases social trust in the safety of drugs.	486 (48%)
Causes legal disputes.	291 (29%)
Heightens social tensions about the risk of drugs.	288 (28%)
Wastes time reporting.	200 (20%)
Increases risk of medical errors.	155 (15%)
Breaks trust with patients.	112 (11%)
Interferes with the medical process.	71 (7%)
Decreases my medical revenue.	68 (7%)
The individual reporting benefits.	67 (7%)
Knowledge for safe use of drugs is accumulated.	798 (78%)

* Answers to question A3: (Which of the following do you agree with are the potential outcomes of SR?) (Multiple selections allowed).

**Table 5:** Reasons for not participating in spontaneous reporting of adverse drug events (multiple selections allowed) (*n* = 489) *.

Choices	No (%)
Not aware of the existence of the SRS.	267 (55%)
Not aware of the reporting procedure.	250 (51%)
Causality is not unclear.	223 (46%)
Clinical severity is not severe.	214 (44%)
Reporting procedure is complicated and inconvenient.	109 (22%)
Not sure what the suspected drug is.	121 (25%)
Concerned that it will be used as grounds for attacking the safety of HM.	102 (21%)
No benefit nor reward for me.	96 (20%)
Not aware of how to assess causality.	95 (19%)
Too much work and not enough time.	94 (19%)
Concerned about legal issues such as lawsuits with patients.	76 (16%)
SR is not my duty.	71 (15%)
HM is not subject to SR.	56 (11%)
It is a prodromal response to healing and therefore not subject to SR.	40 (8%)
Academically useless.	32 (7%)
Patient personal information should not be disclosed.	28 (6%)
Concerned about legal issues such as lawsuits with pharmaceutical companies or suppliers.	28 (6%)
Concerned about personal information of the reporter being disclosed.	26 (5%)

* Answers to question A4 (what are the reasons for not reporting ADEs in the past?) (multiple selections allowed) from 489 respondents who experienced or witnessed adverse drug events (answered yes to question E1) but did not report them to the relevant authorities (answered no to question E2). Abbreviations: HM, herbal medicine; SR, spontaneous reporting; SRS, spontaneous reporting system.

**Table 6:** Measures for activating KMD participation in SR (multiple selections allowed) (*n* = 1021) *.

Choices	No (%)
Simplify reporting procedures.	645 (63%)
Strengthen undergraduate education.	629 (62%)
Establish institutional mechanisms to resolve legal issues of concern.	569 (56%)
Activating post-graduation continuing education and promotion.	549 (54%)
Add the SR of the ADE function to the electronic chart.	504 (49%)
Include HMs in the range of the relief system for adverse drug reactions.	461 (45%)
Providing feedback on reporting results.	402 (39%)
Providing appropriate compensation to the reporters.	375 (37%)
Develop an HM-friendly reporting system.	343 (34%)
KMDs are not required to participate in SR.	16 (2%)

* Answers to question A5 (which of the following measures do you think are appropriate for promoting SR by KMDs?) (allowing for multiple selections) from respondents with no experience of SR. Abbreviations: ADE, adverse drug events; HM, herbal medicine; SR, spontaneous reporting.

**Table 7:** Respondents’ level of experience regarding spontaneous reporting.

Question No.	Questions	Choices	No (%)	*p*-Value
E1	Have you ever experienced or witnessed an ADE following drug administration?	No Yes	498 (49%) 523 (51%)	0.43
E2	Have you ever reported an ADE to the relevant authority?	No Yes	965 (95%) 56 (5%)	<0.01
E3	Have you ever received training on PV, including SRS procedures and causality assessment?	No Yes	928 (91%) 93 (9%)	<0.01
E4	If yes to E3, through which channel did you receive the training (AMS)?	College of KM Another	39 (41%) 57 (59%)	0.07

Abbreviations: ADE, adverse drug event; AMS, allowing for multiple selections; KM, traditional Korean medicine; PV, pharmacovigilance; SRS, spontaneous reporting system.

## Data Availability

Data are available upon reasonable request from the corresponding author.

## Supplementary Materials

The following supporting information can be downloaded at https://www.mdpi.com/article/10.3390/healthcare13131620/s1, Table S1: Sociodemographic factors affecting respondents’ knowledge, attitudes, and experiences of spontaneous reporting.

## Abbreviations

The following abbreviations are used in this manuscript:

ADE	Adverse drug event
AKM	Association of Korean Medicine
CI	confidence interval
HM	Herbal medicine
HMP	Licensed herbal medicinal products
HRM	Herbal raw material
KM	Traditional Korean medicine
KMD	Korean medicine doctor
KMI	Traditional Korean medical institutions
KSDC	Korean Social Science Data Center
MFDS	Ministry of Food and Drug Safety
OR	Odds ratio
PIDM	Programme for International Drug Monitoring
RDSC	Regional drug safety center
SPHM	Self-prepared herbal medicine
SR	Spontaneous reporting
SRS	Spontaneous reporting system
WHO	World Health Organization
WM	Western medicine
